# Irretrievable wire wrap and catheter entrapment: Novel failure mode of endobypass device

**DOI:** 10.1016/j.jvscit.2026.102187

**Published:** 2026-02-17

**Authors:** Shina Patel, Jocelynne T. Dorotan, Kenny Oh

**Affiliations:** Department of Vascular Surgery, Lewis Katz School of Medicine, Philadelphia, PA

**Keywords:** Endovascular intervention, Device complication, Detour endobypass system, Peripheral arterial disease

## Abstract

A 75-year-old man with a history of severe ischemic cardiomyopathy, coronary artery disease status post coronary artery bypass graft, stroke, atrial fibrillation, chronic kidney disease, and recent right metatarsophalangeal amputation was admitted for an ischemic nonhealing wound. Angiography demonstrated right superior femoral artery occlusion with above-knee reconstitution. Percutaneous transmural arterial bypass using the Detour device (Endologix, LLC) was complicated by severe wire wrap and irretrievable catheter entrapment, necessitating open extraction and procedure abortion. Despite technical failure, the wound had healed at follow-up. This case highlights device-specific challenges and divergence between technical and clinical success.

Peripheral artery disease (PAD) is one of the most prevalent conditions seen in the world, affecting more than 230 million people worldwide and 8.5 million adults in the United States.^1^ With its rising incidence, endovascular therapies have revolutionized the management of complex lower extremity arterial occlusive disease by offering less invasive alternatives to open surgery, reducing perioperative risk, and expanding treatment options for high-risk patient populations.^2^

Endovascular management of PAD uses a range of techniques, such as percutaneous transluminal angioplasty, stents, and atherectomy. The Detour endobypass system (Endologix LLC) is a novel endovascular system that creates a bypass through the superficial femoral artery (SFA), into the femoral vein, and back into the distal SFA to offer a percutaneous alternative for long, complex femoropopliteal occlusions not amenable to standard endovascular or surgical approaches. However, its use is limited by the need for suitable venous anatomy, uncertain long-term venous sequelae, and technical complexity, with limited data on durability and reintervention strategies. This case report presents the first documentation of irretrievable wire wrap and catheter entrapment during a Detour endobypass procedure and highlights the need for adaptability when using novel endovascular devices, particularly in high-risk patients. The patient provided written informed consent for publication of the case details and associated images.

## Case presentation

A 75-year-old man with a history of severe ischemic cardiomyopathy (ejection fraction 5%-10%) status post biventricular implantable cardioverter-defibrillator, coronary artery disease status post coronary artery bypass graft, stroke secondary to carotid artery stenosis, paroxysmal atrial fibrillation, aortic stenosis diabetes mellitus, hypertension, and chronic kidney disease stage II presented with 1 month of worsening discoloration and pain of the right second toe with dry gangrene. He underwent open right second metatarsophalangeal amputation with podiatry. Vascular surgery was consulted for noted poor perfusion intraoperatively.

**Fig 1:**
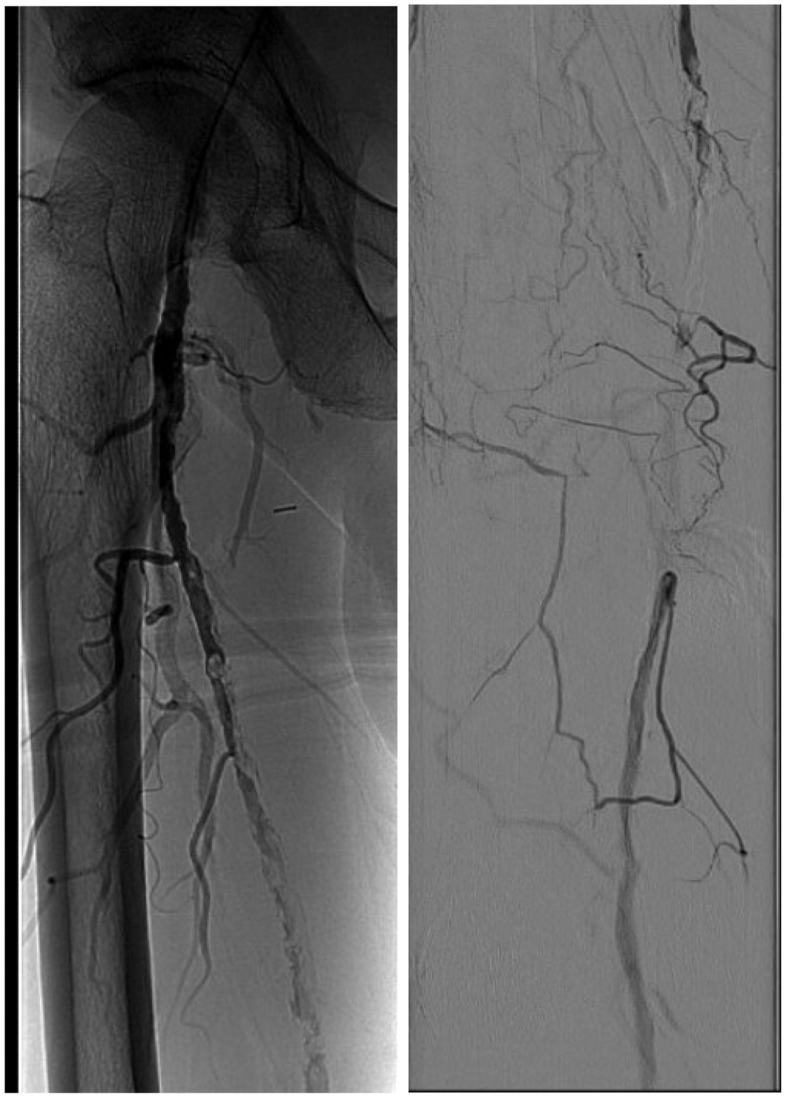
Right lower extremity angiogram demonstrating a diseased superficial femoral artery (left) and reconstitution above the popliteal artery (right).

On physical examination, monophasic Doppler signals were present in the bilateral dorsalis pedis and posterior tibial arteries. A right lower extremity angiogram from 1 month prior demonstrated extensive vascular calcification with anterior tibial/dorsalis pedis and posterior tibial arteries outflowing to the foot, with posterior tibial being dominant. Noninvasive testing showed dampened waveforms and an ankle-brachial index of 0.40. Toe pressures were unable to be obtained due to movement. Vein mapping identified no suitable conduit for surgical bypass. Right lower extremity angiography was performed, which demonstrated a diffusely diseased right SFA, with midsegment occlusion reconstituting at the above-knee popliteal artery and posterior tibial artery-dominant runoff (Fig 1). The posterior tibial and peroneal arteries were patent, with an incomplete plantar arch. Because of possible underfilling, digital runoff could not be appreciated. Given the patient's comorbidities and extensive cardiac history and Revised Cardiac Risk Index score of 5, a multidisciplinary approach was used to optimize the patient's outcomes in line with his goals of limb salvage and proceed with an endovascular procedure.

Before consideration of percutaneous transmural arterial bypass, standard endovascular crossing strategies were attempted. The SFA chronic total occlusion was characterized as a chronic total occlusion crossing approach based on plaque cap morphology class 1 lesion with a tapered proximal cap and above-knee popliteal reconstitution. Antegrade intraluminal and subintimal crossing attempts were made using multiple 0.035″, 0.018″, and 0.014″ guidewires with support catheters but were unsuccessful because of heavy calcification and resistance at the distal cap. Retrograde anterior tibial artery access was attempted without success. Retrograde posterior tibial artery access was considered but deferred because of severe calcification of the dominant runoff vessel. Given failed crossing attempts, lack of bypass conduit, and prohibitive surgical risk, escalation to percutaneous transmural arterial bypass with the Detour system was pursued.

**Fig 2:**
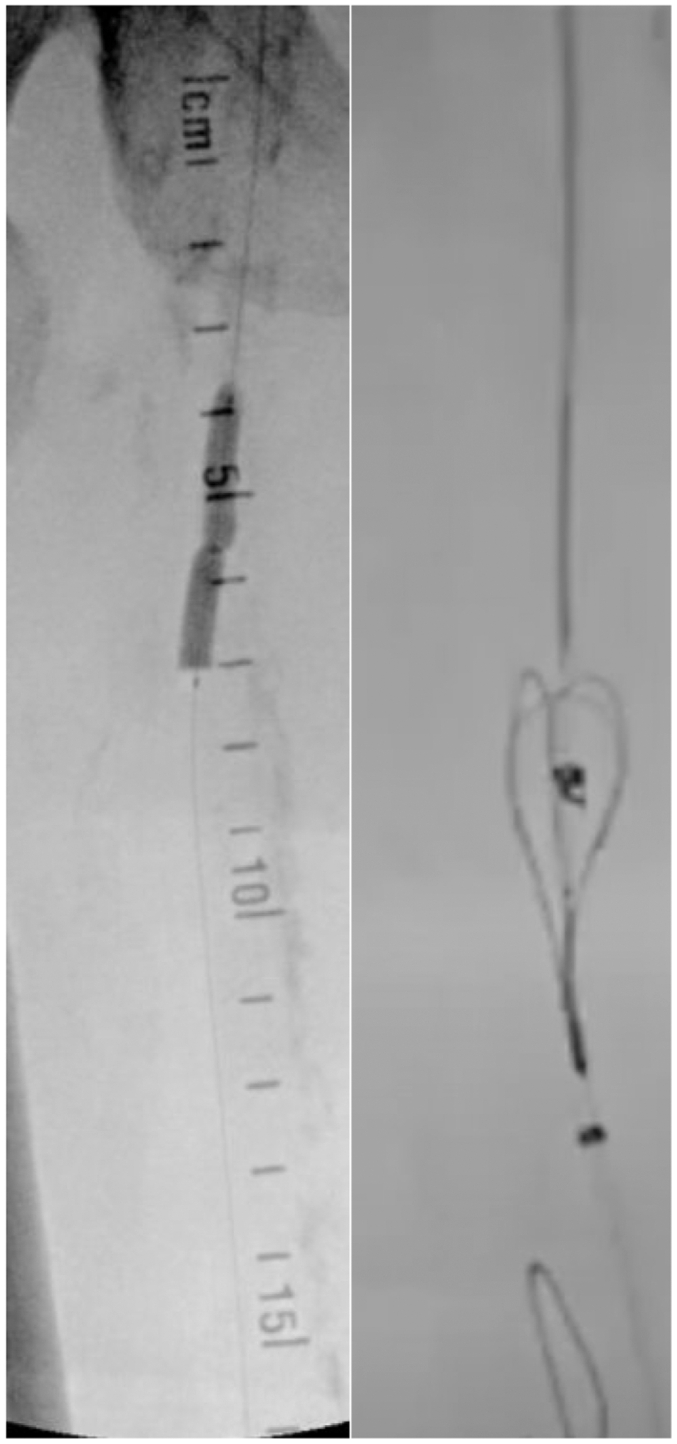
Introduction of the Detour catheter and snaring in the right femoral vein.

At this second angiography procedure, percutaneous right PT vein access was obtained above the ankle. The Detour reentry catheter was introduced from the left common femoral artery access site and tracked to the proximal right superior femoral artery, at which point the re-entry needle was fired and inserted into the femoral vein (Fig 2). The wire was snared from the right femoral vein and externalized through the posterior tibial vein access site, and the proximal arteriovenous connection was ballooned using a 2-mm balloon. The Detour catheter was then advanced further distally in the right femoral vein, where the re-entry needle was fired and re-entered into the P1 popliteal artery. The buddy wire was advanced into the right peroneal artery.

Attempts to retract the re-entry catheter were unsuccessful due to significant wire wrap, which we believe occurred primarily in the extracorporeal portion of the catheter between the buddy wires. This likely resulted from repeated wire advancements, catheter rotations, and multiple re-entry attempts required to navigate the heavily calcified SFA and popliteal arteries, as well as the lack of intravascular ultrasound guidance. To address the entanglement, we attempted removal of each individual arterial wire and performed repeated clockwise and counterclockwise catheter rotations to disentangle the wires; however, all attempts were unsuccessful. Ultimately, a small groin cutdown was performed at the left femoral access site, and Mayo scissors were used to manually unwrap and extract the proximal end of the arterial reentry wire. The procedure lasted approximately 9 hours, with approximately 3 hours spent on routine Detour steps and the remainder devoted to wire wrap salvage and a repeat Detour attempt. No arteriovenous fistula was apparent on completion angiography.

The patient recovered without immediate complications. The patient was seen in clinic 2 months later, at which point the toe amputation site had healed with local wound care and improvement in cardiovascular comorbidities. The ankle-brachial index did not change on follow-up, and the patient remained on aspirin pre- and postoperatively.

## Discussion

This case highlights the complexities and limitations of endovascular revascularization in patients with peripheral arterial disease and significant comorbidities. The Detour endobypass system offers a minimally invasive option for treating long-segment femoropopliteal occlusions, especially in patients who are poor candidates for open surgical bypass due to high operative risk. In this patient, Detour was pursued after standard antegrade, retrograde, and hybrid crossing strategies had failed; he represented a candidate whose SFA chronic total occlusion, despite heavy calcification elsewhere, had accessible proximal and distal entry points and adequate venous anatomy for the procedure. The procedure requires dual access, precise arteriovenous alignment, and coordination of multiple wires and catheters, with technical guidance primarily from preoperative computed tomography and intraoperative fluoroscopy rather than intravascular ultrasound examination.^1^ In this case, repeated wire manipulations and rotations—particularly at the P3 popliteal artery re-entry site—likely contributed to wire wrap. Without pre-existing popliteal access, vessel preparation options such as lithotripsy were not feasible. Although clinical trials such as Detour 2 report high technical success (96%–100%) and acceptable short-term outcomes, our experience reveals previously unreported failure modes and underscores the need for careful patient selection and operator awareness of device limitations.^2^, ^3^, ^4^

This case also highlights operator- and procedure-specific considerations. The team was early in the learning curve for the Detour system, performing one of their first three cases with the device, which may have contributed to challenges in catheter manipulation. The procedure was also performed under conscious sedation in a patient with significant cardiac comorbidities, emphasizing that even minimally invasive interventions carry substantial physiologic risk, especially if prolonged. Extended sedation increases exposure to hemodynamic fluctuations, hypotension, and bradycardia, particularly in patients with reduced ejection fraction or advanced coronary disease.^5^ Complex technical maneuvers requiring sustained immobility further elevate risk, highlighting the importance of careful preprocedural planning, anesthetic strategy, and consideration of general anesthesia for prolonged, high-risk interventions.

The patient's toe amputation site healed despite abortive revascularization, highlighting the role of collateral perfusion and patient-specific factors in compensating for technical failure. ABI remained unchanged on follow-up; however, optimization of cardiovascular medications and a mild increase in ejection fraction may have supported tissue perfusion and contributed to wound healing. Chronic ischemia triggers arteriogenesis and vascular endothelial growth factor-mediated angiogenesis, partially maintaining distal perfusion even without successful macrorevascularization.^6^ Although successful revascularization typically improves long-term cardiovascular outcomes, patient-centered end points—such as wound healing, pain control, and function—can still improve after partial or unsuccessful procedures.^7^ This case underscores that the decision to pursue revascularization should be individualized, balancing procedural risk with patient physiology and goals.

## Conclusions

By documenting a previously unreported failure mode—wire wrap with retained re-entry catheter—during the Detour procedure, this report expands on the understanding of the limitations of percutaneous transmural arterial bypass. Publishing such experiences is essential to refine device design, operator technique, and complication management strategies, ultimately improving safety and outcomes in high-risk PAD interventions.

## Funding

None.

## Disclosures

None.
